# Supplementary material for: Presence of Ceramidase Activity in Electronegative LDL
Source: Int J Mol Sci. 2022 Dec 22;24(1):165. doi: 10.3390/ijms24010165 (PMC9820682; doi:10.3390/ijms24010165)
Supplement: Supplementary file 1 [file ijms-24-00165-s001.zip › ijms-1927860-supplementary.pdf]

Supplementary Material

Presence of ceramidase activity in electronegative LDL

Núria Puig[1,2], Jose Rives[1,2], Montserrat Estruch[3], Ana Aguilera-Simon[4], Noemi Rotllan[5,6], Mercedes Camacho[7,8], Núria Colomé [9], Francesc Canals[9], José Luis Sánchez-Quesada[1,6,*], and Sonia Benitez[1,6,*]

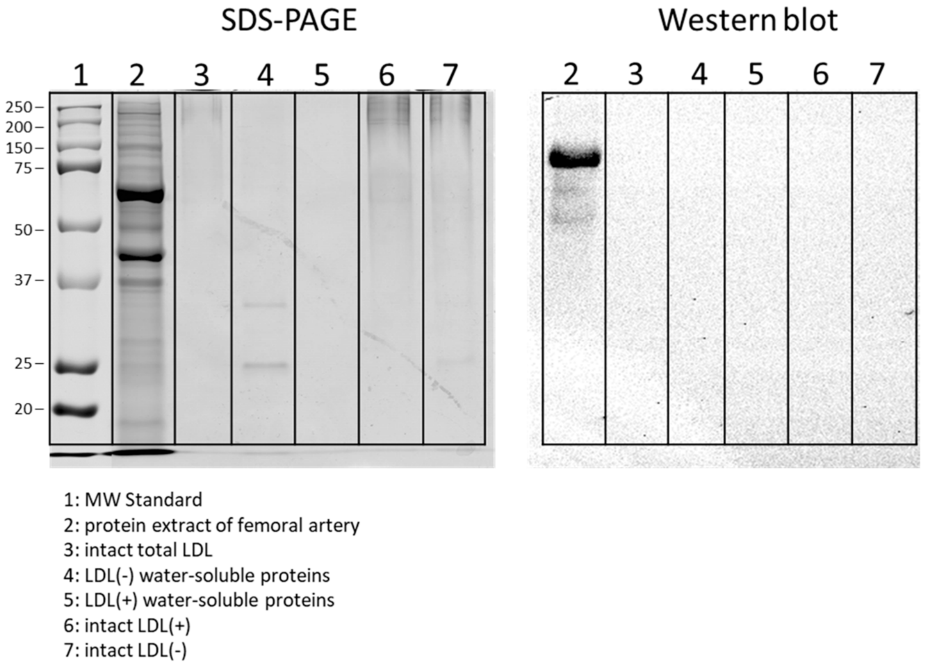

Supplementary Figure S1. Western blot analysis of LDL subfractions to detect neutral ceramidase.

The putative presence of neutral CDase in LDL(-) was assessed by western blot. Intact total LDL (10 μg/lane), LDL(+) (20 μg/lane), and LDL(-) (20 μg/lane), as well as the moeties of water-soluble proteins from LDL(-) and LDL(+), were run in 10% SDS-PAGE gels, as described below. A protein extract of femoral artery was used as a positive control.

Supplementary Methods

Delipidation of LDL subfractions

LDL subfractions (150 μg apoB) were delipidated according to Bligh and Dyer method (Bligh EG, Dyer WJ. Can J Biochem Physiol. 1959 Aug;37(8):911-7). The upper phase contains the low-sized water-soluble proteins, which were assayed for western blot

analysis. This phase was concentrated 10-fold using centrifugation concentrators (Amicon, 10kDa MWCO).

Western blot of neutral CDase

Electrophoresis was performed at 100 V for 2 h at room temperature. One gel was stained with Coomassie Brilliant Blue. Proteins of the other gel were transferred to a PVDF membrane (2 h at 30 V, in Tris-glycine buffer containing 20% methanol and 0.1% SDS) and blocked for 1 h at room temperature with blocking buffer (Bio-Rad). Western blot was performed using primary mouse monoclonal antibody IgG anti-nCDase (B9 sc374634, Santa Cruz Biotechnology) and secondary HRP-conjugated anti-mouse IgG (m-IgG2b BP-HRP, Santa Cruz Biotechnology). PVDF membrane was incubated overnight at 4ºC with IgG anti-nCDase diluted 1/100 in blocking buffer, washed 3-fold with TBS containing 1% tween 20 (TTBS) and then incubated with the secondary antibody (dilution 1/1000 with TTBS) for 2 hours at room temperature. PVDF was revealed with Clarity western ECL substrate kit (BioRad).
